# Do we really need standards in digital image management?

**DOI:** 10.2349/biij.4.1.e20

**Published:** 2008-10-01

**Authors:** ELM Ho

**Affiliations:** College of Radiology, Academy of Medicine of Malaysia

**Keywords:** Digital image management, DICOM, PACS, lossy compression, lossless compression

## Abstract

Convention dictates that standards are a necessity rather than a luxury. Standards are supposed to improve the exchange of health and image data information resulting in improved quality and efficiency of patient care. True standardisation is some time away yet, as barriers exist with evolving equipment, storage formats and even the standards themselves. The explosive growth in the size and complexity of images such as those generated by multislice computed tomography have driven the need for digital image management, created problems of storage space and costs, and created a challenge for increasing or getting an adequate speed for transmitting, accessing and retrieving the image data. The search for a suitable and practical format for storing the data without loss of information and medico-legal implications has become a necessity and a matter of ‘urgency’. Existing standards are either open or proprietary and must comply with local, regional or national laws. Currently there are the Picture Archiving and Communications System (PACS); Digital Imaging and Communications in Medicine (DICOM); Health Level 7 (HL7) and Integrating the Healthcare Enterprise (IHE). Issues in digital image management can be categorised as operational, procedural, technical and administrative. Standards must stay focussed on the ultimate goal – that is, improved patient care worldwide.

## INTRODUCTION

Does the theory of standardisation translate into truly useful day-to-day clinical practice that all involved in the digital imaging era can relate to? What barriers are we facing, and how do we overcome them?

Standard or Standards by definition is the level of quality where people think is acceptable (Oxford Advanced Learner’s Dictionary). This may refer to behaviour where the morally acceptable level is the standard or an official unit of measure. For example, the industry standard may refer to a specific size and the gold standard is what others are compared to. Standard could also mean what is normal or average for a person, situation or even a language. When standards are applied, quite often there are modifications because there is a need to be ‘different’, to stand out from the rest, to have better display and to communicate an idea or message better.

In the setting of standards, who should be the stakeholders? For digital image management, this could include, in no specific order, the scientists, engineers, inventors, hardware specialists, software programmers, communications specialists, vendors, marketing personnel, the users (for example, the radiologists and radiographers) and the government.

## ISSUES IN DIGITAL IMAGE MANAGEMENT

The explosive growth in the size and complexity of images such as those generated by multislice computed tomography (MSCT), dual source computed tomography (DSCT) and positron emission tomography–computed tomography (PET-CT) have driven the need for image management, created problems of storage in terms of space and costs and created a challenge for increasing or getting an adequate speed for transmitting, accessing and retrieving the image data. Previously a CT scan would generate 40-60 images, now it easily goes into 900 images. Magnetic resonance imaging (MRI) angiography or cardiac MRI could have images totalling 15,000 for a larger scan series. With the great improvements and innovative imaging equipment development, the bottleneck is now in the time required for reconstruction of complex datasets and the time to process the images for display and interpretation.

Storage issues include how to compress, how much compression and the size after compression. Compressing thin slices (as in the multislice CT at 0.75mm) is more difficult because there will be inherently more noise on the data, yet with less redundant information.

As in all electronic data storage, backups are imperative and management includes ensuring reliability and redundancy for breakdowns in the system. Then there are security and privacy issues, litigation and laws which may vary from country to country. Other considerations include patient details, reports and other relevant and related clinical information, the need to link to other centres and the financial costs.

We can categorise issues in digital image management as operational, procedural, technical and administrative. We are therefore looking at compatibility, interchangeability and interoperability.

## WHAT SHOULD THE STANDARDS DEFINE?

The American College of Radiology (ACR) Technical Standard for Digital Image Data Management [1] specifies the following: goals, personnel qualifications, equipment guidelines, specifications of data manipulation and management, quality control and quality improvement methods.

Goals of digital image management as expressed in the ACR Technical Standards for Digital Image Data Management include accurate labelling and identification of image data in the acquisition, generation and recording of image data, transmission of images to appropriate storage medium for retrieval for display and formal interpretation, review and consultation. Retrieval of available prior imaging studies is essential for comparison with current studies. Images should also be able to be transmitted to remote sites for consultation, review or formal interpretation. There should be appropriate image data compression to facilitate storage or transmission without loss of clinically significant information. Archived data should contain accurate patient medical records for timely retrieval, and must meet applicable facility, state and other regulations while maintaining patient confidentiality.

As compression formats are still being developed and tested while imaging modalities are improving and enabling more functions, goals of digital image management will also be evolving. We also need to integrate advanced image processing into the system (3-dimensional as well as computer aided detection).

For equipment specifications, compliance with the American College of Radiology (ACR)- National Electrical Manufacturers Association (NEMA) Digital Imaging and Communications in Medicine (DICOM) [2] standard is strongly recommended for all new equipment acquisitions and consideration of periodic upgrades.

## BARRIERS TO TRUE STANDARDISATION

These barriers include the fact that goals, image acquisition, compression formats, storage systems, and communications are all still evolving. Add to this the legacy systems and the fact that equipment vendors are competing to develop better imaging equipment and ‘one-up’ each other in marketing. Then, one must look at the other side of the coin: are standards going to make the situation rigid and stifling? Will it stem creativity, innovation, improvements and progress?

Needless to say, since utopia has yet to be attained, things must continue and need to evolve. So it is with foresight that the ACR technical standard preamble includes the following – that ‘the technical standard is just an educational tool to assist practitioners in the provision of appropriate radiologic care and it is not a set of inflexible rules or requirements of practice and is also not intended nor should be used to establish a legal standard of care’ [1].

## STANDARD OR STANDARDS ALREADY IN EXISTENCE

Standards can be open or proprietary. Currently there are the Picture Archiving and Communications System (PACS); Digital Imaging and Communications in Medicine (DICOM); Health Level 7 (HL7) and Integrating the Healthcare Enterprise (IHE). These shall only be dealt with in brief.

PACS was developed to provide an organised mechanism for digital image management. There are single modality PACS, minipacs or multimodality PACS. An image management specialist is needed for the PACS. This system may become the standard in hospitals within the next decade in North America and United Kingdom as well as Nordic countries. The increased utilisation is due to new digital imaging modalities, reduced costs (thanks to web-based solutions, affordable software licences, reduced costs of flat panel displays and storage) and government-driven initiatives, for example, Britain’s National Health Service.

As mentioned earlier, the number of images generated has increased exponentially, such that there is really no choice but to adopt PACS. In a properly implemented PACS, radiologists can see up to 10% more patients per day, perform post processing, 3D rendering and surgical planning with PACS, and access images from home.

DICOM [2] is also an evolving standard and facilitates PACS development. It allows creation of diagnostic information databases that can be accessed by a variety of devices worldwide. The DICOM standard is a structured multipart document and arose as there was a need to transfer images and associated information between devices manufactured by various vendors. These devices produced a variety of digital image formats!

HL7 [3] is a standard for exchanging information between medical applications and is just a protocol for data exchange. It defines the format and content of messages to be used when exchanging data in various circumstances. It promotes the use of such standards within and among the healthcare organisations to increase the effectiveness and efficiency of healthcare delivery for the benefit of all.

IHE [4] is a multi-year initiative that creates the framework for passing vital health information seamlessly from application to application, from system to system and from setting to setting across the entire healthcare enterprise. IHE is under the leadership of the Healthcare Information and Management Systems Society (HIMSS) and the Radiological Society of North America (RSNA). It has been around since 1998. Before the IHE, there was no agreed method for various systems in Radiology to work together – HIS (Hospital Information System), RIS (Radiology Information System), PACS, printers, workstations and various imaging equipment. There are at least 16 IHE Radiology Integration Profiles.

## WHERE TO FROM HERE?

In a recent report, researchers at the Dana-Farber Cancer Institute in Boston [5] developed a solution in the form of a PET/CT database of all settings of prior imaging procedures to allow consistent imaging of cancer patients over time. This was necessary as current PACS and HIS/RIS did not capture this data. This illustrates how and why current digital image management systems and standards will need to evolve.

Lossy (irreversible compression with some loss of information) or Lossless (reversible compression with no loss of information) – that is the question [6]? How to compress and compression to what size are questions that are still under study. The litigation potential of missed or inaccurate diagnosis of irreversibly compressed images is a major factor contributing to why equipment makers will delay adoption of newer compression formats. Loss of information, of course, concerns radiologists, patients and other physicians. The concern with lossy compression is that the reconstructed image quality may be affected and there may be perceived or actual distortion of clinically significant image details.

In lossless compression, the decompressed image is numerically identical to the original. Examples of lossless compression include run-length encoded (RLE), low ratio JPEG (Joint Photographic Experts Group) and JPEG-LS (the new JPEG lossless compression standard). Currently the focus of some groups is determining if lossy image compression can be used in Radiology without compromising information for interpretation. The DICOM working group 4 in 2002 has already announced the wavelet-based JPEG 2000 compression algorithm as standard. JPEG 2000 has higher compression with less distortion. No diagnostic data is discarded during the compression although some data will be discarded during compression and cannot be recovered.

The ACR Technical Standard for Digital Image Data Management [1] does not specify an acceptable compression ratio – and this is left to the discretion of a qualified physician. The Canadian Association of Radiologists (CAR) PACS and Teleradiology Committee has accepted lossy compression for use in primary diagnostic and clinical review. Compression ratios may differ depending on the imaging modality and for different organ systems within a single modality. For example, a musculoskeletal image can be compressed to a greater degree than a chest image. It is also of interest to note that images compressed with JPEG 2000 at low ratios may actually have better quality than original images. This was attributed to the first level of decomposition in wavelet compression, which at low filter eliminates noise and therefore improves visual quality.

## THE PRACTICE OF STANDARDS IN REALITY

There is no reason to doubt that everyone wants to support standards. Standards in general are supported, adopted and may or may not be used in its entirety. It all depends on the 'local' requirements, available infrastructure, resources (which include human and financial), the local/regional laws, and the need for security and privacy. We note the need for the ‘birth’ of new personnel – the image management specialist [1]. This person has to assess and provide problem solutions, initiate repair, coordinate system-wide maintenance, be available in a timely manner for trouble shooting or malfunction correction, and be directly involved in system expansion to assure sustainable high image quality and system function.

In reality, standards are actually slow to garner support, and slow to be adopted. Even more questionable would be to what extent these standards are used. Technophobia is not uncommon amongst all categories of users, including the radiologists. The image acquisition vendor may also be ‘lethargic’ as in the case of adopting enhanced DICOM objects. Enhanced DICOM objects were added to the DICOM standard in 2003. PACS vendors and CT vendors were slow to support and adopt this, respectively. JPEG2000 shows great promise in lossy compression of thin section data, yet this fact has not been taken advantage of mainly because of medico-legal considerations.

## DIGITAL IMAGE MANAGEMENT – AN EXPENSE OR AN INVESTMENT?

Is digital image management an expense or an investment? Adequate capital is needed for systems such as PACS. If there is still a need to print on films, then there is increased cost and negates some of the plus points in using PACS. Training costs are involved and there is a learning curve for users. If users cannot be motivated to see how it will help them in their daily routines, learning and acceptance will be uphill tasks. There is also a need to employ more personnel for technological and technical support.

Investments should support business needs, and proposals to adopt PACS/digital image management need to be presented in a manner where the hospital or department management can see returns or benefits. It is more important to define the value of the project over its entire life rather than just ‘returns on initial investment’. The payback (how soon the investment will be recovered) and the opportunity costs (the cost of passing up the next best choice when making a decision) as well as soft benefits such as qualitative measures of productivity, image and morale will all assist in the successful bid for the hospital budget and enable proper implementation.

Challenges in implementation will exist but the satisfaction comes in a properly implemented and well-thought out digital image management system.

## CONCLUSION

Standards in digital image management are needed. The various issues, although easily categorised into operational, procedural, technical and administrative, will entail far more in practical terms. All the stakeholders – the scientists, engineers, inventors, hardware specialists, software programmers, communications specialists, vendors, marketing personnel, the users such as the radiologists and radiographer and governments – need to cooperate in recognising and evaluating the evolving needs and respond with flexibility and agility in the development of standards. The ultimate goal is improved quality and efficiency of patient care worldwide through improved exchange of health and image data information and improved access in remote areas.
